# Study protocol for WHO and UNICEF estimates of global, regional, and national preterm birth rates for 2010 to 2019

**DOI:** 10.1371/journal.pone.0258751

**Published:** 2021-10-20

**Authors:** Ayesha De Costa, Ann-Beth Moller, Hannah Blencowe, Emily White Johansson, Laith Hussain-Alkhateeb, Eric O. Ohuma, Yemisrach B. Okwaraji, Jennifer Cresswell, Jennifer H. Requejo, Rajiv Bahl, Olufemi T. Oladapo, Joy E. Lawn, Allisyn C. Moran

**Affiliations:** 1 Department of Maternal, Newborn, Child and Adolescent Health and Ageing, World Health Organization, Geneva, Switzerland; 2 UNDP-UNFPA-UNICEF-WHO-World Bank Special Programme of Research, Development and Research Training in Human Reproduction (HRP), Department of Sexual and Reproductive Health and Research World Health Organization, Geneva, Switzerland; 3 Centre for Maternal, Adolescent, Reproductive and Child Health, London School of Hygiene and Tropical Medicine, London, United Kingdom; 4 Department of Women and Children’s Health, Uppsala University, Uppsala, Sweden; 5 Global Health, School of Public Health and Community Medicine, Institute of Medicine, Sahlgrenska Academy, University of Gothenburg, Gothenburg, Sweden; 6 Division of Data, Analysis, Planning and Monitoring, United Nations Children’s Fund, New York City, New York, United States of America; University of Mississippi Medical Center, UNITED STATES

## Abstract

**Background:**

Preterm birth is a leading cause of death among children under five years. Previous estimates indicated global preterm birth rate of 10.6% (14.8 million neonates) in 2014. We aim to update preterm birth estimates at global, regional, and national levels for the period 2010 to 2019.

**Methods:**

Preterm birth is defined as a live birth occurring before 37 completed gestational weeks, or <259 days since a woman’s last menstrual period. National administrative data sources for WHO Member States with facility birth rates of ≥80% in the most recent year for which data is available will be searched. Administrative data identified for these countries will be considered if ≥80% of UN estimated live births include gestational age information to define preterm birth. For countries without eligible administrative data, a systematic review of studies will be conducted. Research studies will be eligible if the reported outcome is derived from an observational or intervention study conducted at national or sub-national level in population- or facility-based settings. Risk of bias assessments will focus on gestational age measurement method and coverage, and inclusion of special subgroups in published estimates. Covariates for inclusion will be selected a priori based on a conceptual framework of plausible associations with preterm birth, data availability, and quality of covariate data across many countries and years. Global, regional and national preterm birth rates will be estimated using a Bayesian multilevel-mixed regression model.

**Discussion:**

Accurate measurement of preterm birth is challenging in many countries given incomplete or unavailable data from national administrative sources, compounded by limited gestational age assessment during pregnancy to define preterm birth. Up-to-date modelled estimates will be an important resource to measure the global burden of preterm birth and to inform policies and programs especially in settings with a high burden of neonatal mortality.

**Trial registration:**

**PROSPERO registration:** CRD42021237861.

## Introduction

Preterm birth (birth occurring before 37 completed weeks of gestation) is a leading cause of death in children under five years of age globally. In 2019, preterm birth accounted for approximately 16% of deaths in this age group and 35% of neonatal deaths [1]. Besides mortality, complications from preterm birth are also an important cause of serious morbidity associated with prolonged hospital admission for respiratory, metabolic, neurological and infectious morbidities [2–10]. Preterm birth has also been shown to be associated with components of the metabolic syndrome and cardiovascular disease in adult life [11,12]. Preterm birth is therefore associated with significant costs to health systems [13–16].

Achieving significant declines in neonatal deaths will require strong commitment to the reduction of mortality among preterm newborns. Estimates of the burden of preterm birth are therefore critical to plan appropriate strategies to reduce preterm birth-related mortality and morbidity, as well as for achieving Sustainable Development Goal (SDG) 3.2 [17], which aims to end preventable deaths of newborns and children under five years of age by 2030. This target further specifies that all countries, by 2030, should aim to reduce neonatal mortality to at least as low as 12 per 1,000 live births. Given that preterm birth is a major contributor to neonatal and child mortality [1], reducing preterm birth is central to achieving SDG 3.2.

Yet, the accurate measurement of preterm birth has been challenging in many countries given incomplete or unavailable data from national administrative systems. Data constraints are further compounded by limited ascertainment of gestational age during pregnancy, particularly in low-resource settings, which is critical to classify a birth as preterm. The improved measurement of preterm birth has therefore been highlighted as an important priority of the *Every Newborn Action Plan* [18], led jointly by the World Health Organization (WHO) and the United Nations Children’s Emergency Fund (UNICEF). The development of these estimates also serves to emphasize the need for strengthening administrative data systems to report national preterm birth rates.

Given these data constraints and the ongoing need to strengthen national data reporting on preterm births, it is necessary to develop comparable model-based estimates of preterm birth at global, regional and national levels, while at the same time continuing to support improved national administrative data systems that will help to improve preterm birth measurement in the future.

Three analyses of the global burden of preterm birth have been published to date [19–21]. The most recent analysis showed that 14.8 million (uncertainty interval [UI]: 12.7–16.7 million) were born preterm worldwide in 2014 [21]. Approximately 80% of these preterm births were estimated to have occurred in South Asia and sub-Saharan Africa.

Global estimates of preterm birth require regular updates to enable the inclusion of new data and to best reflect current levels and trends. Up-to-date global preterm birth estimates are crucial to understand the recent epidemiology of preterm birth and its variations across regions and countries; to support the development and implementation of policies for newborn health; to inform resource allocation within health systems; and to aid impact assessments of interventions to promote newborn health. These estimates will also help to raise awareness about preterm birth as a leading global public health challenge and will be critical for highlighting the need for continued investments in national administrative data systems to improve preterm birth measurement and reporting in the future. Finally, the current round of preterm birth estimates will be the first to cover the beginning of the SDG era, and will also inform the Survive, Thrive, Transform agenda of the Global Strategy for Women’s, Children’s and Adolescents’ Health (2016–2030) [22].

### Objectives

The main objective of this protocol is to describe the methodology for the data compilation and the statistical modelling that will be applied to develop the fourth round of global estimates for preterm birth for the period 2010 to 2019, with associated uncertainty intervals.

## Methods

### Project organization

A Steering Group, comprised of experts from the WHO, UNICEF and the London School of Hygiene and Tropical Medicine (LSHTM), will implement this protocol. The work will be supported by an Estimates Consultative Group (ECG), which is comprised of global experts in preterm birth measurement including obstetricians, neonatologists, statisticians, preterm birth researchers, and program staff working in the measurement field. The ECG will provide technical guidance on the estimation process, as well as review data inputs and preliminary estimates prior to finalization. An official country consultation will be conducted with WHO Member States to inform Member States of the methodology [23], as well as to invite review of preliminary national estimates and to share additional data not already identified for consideration in this work.

### Preterm birth definition

The International Statistical Classification of Diseases and Health Problems, 11^th^ revision (ICD-11) uses the WHO definition of preterm birth, namely: “*All births before 37 completed weeks of gestation or fewer than 259 days since the first day of a woman’s last menstrual period*” [24]. The WHO definition does not define a lower gestational age limit for reporting, and ICD-11 further advises inclusion of all live births (regardless of gestational age).

WHO recommends reporting the preterm birth rate using the following indicator:

$$Preterm\;birth\;rate=\frac{Number\;of\;live\;born\;preterm\;births\;(singleton\;or\;multiple)\;x\;100\%}{Number\;of\;live\;births\;(single\;or\;multiple)}$$

The primary outcome for this study is therefore defined as a live birth occurring before 37 completed weeks of gestation, or fewer than 259 days since the woman’s last menstrual period. The secondary outcome includes preterm birth based on different gestational age ranges including: (1) extremely preterm (<28 completed weeks of gestation); (2) very preterm (28 to <32 weeks of gestation); (3) moderately preterm (32 to <34 weeks of gestation); and late preterm (34 to <37 weeks of gestation).

Gestational age measurement to classify a preterm is limited in many countries. Furthermore, the method used to define a preterm birth may substantially impact reported national rates (S1 Appendix). Ultrasound dating of gestational age during the first trimester is the ‘gold standard’ [25]. Yet access to early pregnancy ultrasound is limited in many countries, and these settings may rely instead on less accurate measures such as last menstrual period (LMP), symphysis-fundal height, post-natal examination, or ultrasound scan in later pregnancy stages (see “Risk of Bias Assessment” section).

### Data sources

**Fig 1:**
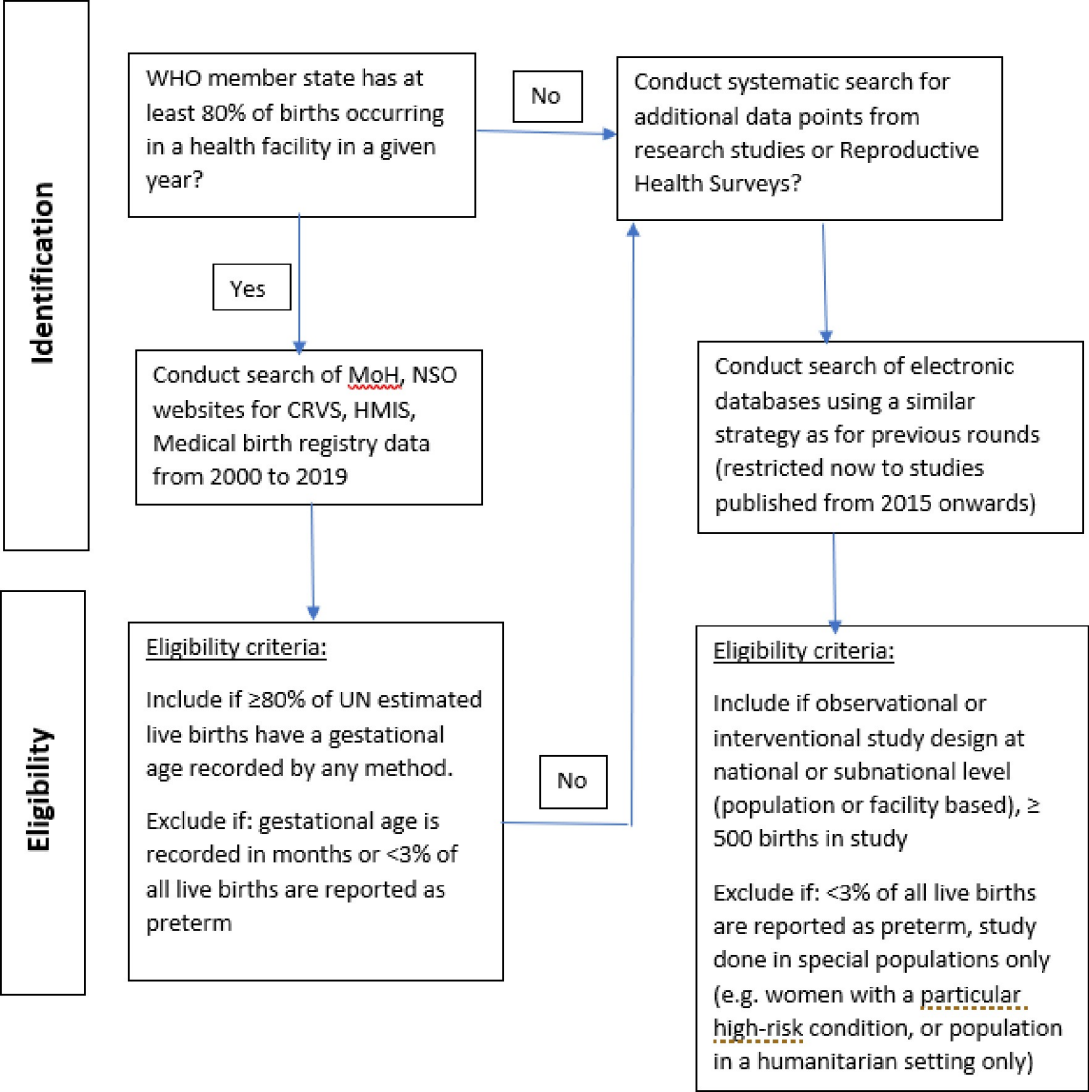
Flow diagram of the data search and review process.

There are two broad categories of data sources that may be used for preterm birth estimation; national administrative data and research studies including Reproductive Health Surveys [26]. National administrative data based on Civil Registration and Vital Statistics (CRVS), Health Management Information Systems (HMIS), and Medical Birth Registries are the preferred data sources for preterm birth rates. However, for many countries, data from administrative sources are incomplete or not available [27]. For these countries, a systematic review of research studies, including Reproductive Health Surveys, will be undertaken to identify additional data points that may be used in the estimation process. Fig 1 summarizes the search strategy and eligibility criteria for the systematic review, which is further described in the below sections.

### Search strategy

For administrative data sources, we will conduct a systematic search of Ministry of Health and National Statistical Office [28] publications and datasets for WHO Member States that have a population-based facility birth rate of at least 80% in the latest year for which data is available between 2010 and 2019 [28,29]. For countries that meet this threshold, administrative data sources used in the previous round will initially be searched to identify more recent data points. For countries without administrative data from previous rounds, a systematic search of Ministry of Health, National Statistical Office, or other national perinatal databases will be conducted. Data points from the previous estimation round (2000–2014), combined with those obtained from the current systematic review (2015-present), will be used to inform this latest round of estimates for the period 2010–2019. The search terms used for English-language websites will include: *birth*, *gestational age*, and *preterm* (with appropriate translations for non-English websites). Non-English websites will be searched by researchers who speak the relevant language.

A systematic review of studies will be conducted for WHO Member States that do not meet the threshold to search administrative data sources and that lack eligible administrative data for the estimation work (see “Eligibility Criteria"). We will search the following electronic databases: MEDLINE, EMBASE, WHO Global Index Medicus, CINAHL, PsycINFO, and the Cochrane Central Register of Controlled Trials (CENTRAL). All searches will be restricted to publication dates after 1 January 2015 in order to update the database from the previous estimation round [30]. There will be no language restrictions in the search. The search strategies used are provided in the appendix (S2 Appendix). Studies identified in the search will be imported into Covidence software for screening, review and data extraction if eligibility criteria are met [31].

### Eligibility criteria

For administrative data sources, all CRVS, HMIS or Medical Birth Registry data identified through the search will be eligible for inclusion if at least 80% of the United Nations (UN) estimated live births in a given country for a given year are reported with gestational age information to define preterm birth [32]. For administrative data sources, there are no eligibility restrictions on the method of gestational age measurement used to classify a preterm birth since this information may not be reported in CRVS, HMIS or Medical Birth Registries.

For studies, all data sources identified through the search will be eligible for inclusion if the outcome is derived from an observational or intervention study design conducted at national or sub-national level in either population- or facility-based settings. Study designs may include randomized controlled trials, non-randomized trials, cross-sectional studies, controlled before-after studies, or longitudinal studies with a sample size of least 500 births. For intervention studies, outcomes reported for the control arm will be used. In addition, for research studies, eligible gestational age measurement methods to classify a preterm birth may include: ultrasound in any trimester, last menstrual period, symphysis-fundal height, post-natal assessment, or any combination of these measures (see “Risk of Bias Assessment” section).

### Data screening and review

For administrative data, all CRVS, HMIS and Medical Birth Registry data identified through the search will be reviewed against eligibility criteria. For research studies, all titles and abstracts of identified studies will be independently screened by two reviewers for potential eligibility and, if disagreements occur, the full text will be reviewed. The full text of potentially eligible studies, plus studies where disagreements occur, will be retrieved and independently assessed by the same two reviewers. Any disagreements during the full-text review stage will be discussed to reach consensus or will be settled through consultation with a third reviewer. Exclusion reasons will be documented for the full-text review stage.

### Data extraction

For all eligible data sources, the following data points will be extracted: country, data source type, study design type (if applicable), time period, citation or website, outcome definition, method for gestational age measurement, gestational age range(s), total preterm births, total live births, and any covariate data.

For administrative data sources, data will be extracted into an excel-based form (S3 Appendix) as part of the review process using the process previously described. For research studies, data will be extracted by two independent reviewers into Covidence software using the same procedures for screening and review. For non-English data sources, the review and data extraction process will be supported by researchers who speak the relevant language.

### Exclusion criteria

All data sources reporting gestational age in months or where less than 3% of live births are reported to be preterm will be excluded. This cut-off is aligned with the lowest proportions of preterm birth (3.4%) reported for countries by the Intergrowth 21^st^ project [33].

For administrative data only, datasets that provide gestational age information for less than 80% of UN estimated live births in a given country for a given year will be excluded;

For research studies only, the following additional exclusion criteria will be applied:

- Study populations derived from high-risk sub-national groups will be excluded. For example, studies including only women with specific medical or obstetric complications; studies based on other high-risk sub-national populations only (e.g. sub-national humanitarian settings only; indigenous populations only).
- Case-control studies will be excluded due to the potential for selection bias.

### Risk of bias assessment

Assessing the risks of bias informs the extent to which the reported preterm birth prevalence observed in the data source may differ from the “true” prevalence at the national level. **Table 1** describes the potential sources of bias which focus on the coverage and method of gestational age measurement, and the representativeness of the liveborn neonate population from which the outcome is derived.

**Table 1:** Risk of bias assessment.

Source of bias	Rationale	Categories	Proposed mitigation
Coverage of gestational age measurement to define preterm birth	Better coverage of GA measurement more likely in high-income settings where preterm birth rates are usually lower.This could bias the estimates downward.	<80%, 80–89%, or at least 90% of UN estimated live births (or study population) with gestational age data to define preterm birth	This criterion may be used in sensitivity analyses. Data points with <80% of births with a gestational age will be excluded
Method of gestational age measurement	First trimester ultrasound is considered ‘gold standard’. Other measures can contribute to biases in either direction due to lower accuracy (S1 Appendix).	Ultrasound (<14, 14–24, or >24 weeks); last menstrual period; symphysis-fundal height; or unknown	This criterion may be used in sensitivity analyses.
Study participants (or administrative data source) include disadvantaged groups within the country	Disadvantaged populations within a country generally have higher preterm birth rates. Lack of their inclusion in data sets could bias the estimates downward.	Yes, No, Not Reported	This will be reported descriptively, and the implications discussed.

For administrative data sources, the biases will be assessed as part of data extraction phase. For eligible studies, two independent reviewers will conduct the assessment. Any disagreements will be discussed to reach consensus or will be settled through consultation with a third reviewer. The results of the risk of bias assessment will inform statistical analyses through potential inclusion as model inputs or sensitivity analyses and will also be used to interpret results and discuss potential study limitations.

### Data management

The database from previous estimation rounds will be updated with newly eligible data points from the current systematic searches and review. If there are duplicate data points, the latest extracted data point will be maintained in the database. All data points from studies extracted into Covidence [31] will be exported into the excel-based data extraction form to create one complete database with both administrative and research study data points.

### Statistical analysis

#### Step 1: Covariates to be considered

**Fig 2:**
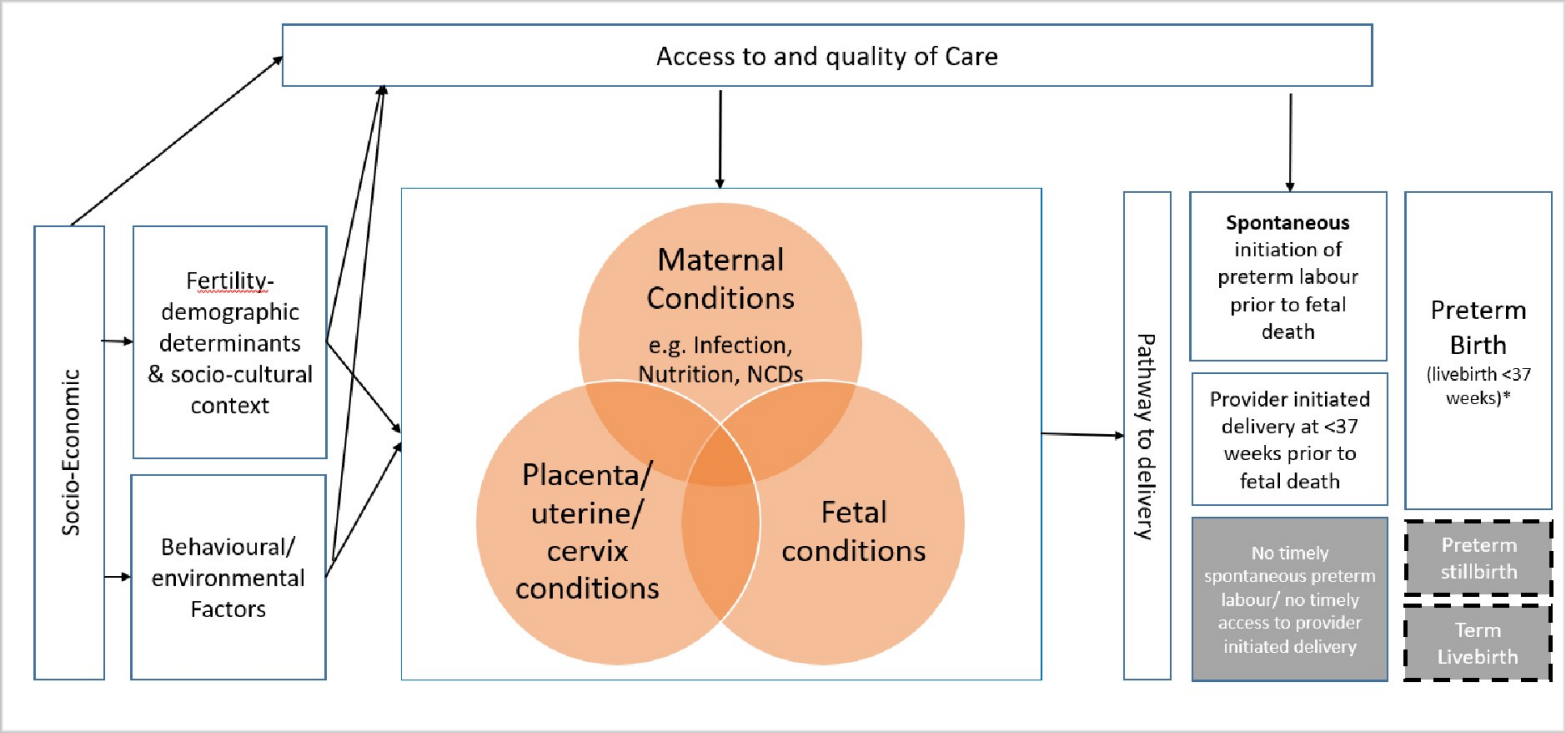
Conceptual framework for covariate selection. Note: Adapted from previous conceptual frameworks [34–36].

The development of the models for the preterm birth estimates will utilize country-level covariates from available United Nations and other sources. Covariates for inclusion will be selected a priori, based on the conceptual framework of plausible associations with preterm birth, data availability, and quality of covariate data. Fig 2 presents a conceptual framework illustrating the pathways to preterm birth and the relationship between socio-economic and demographic factors, maternal health status (e.g. infections and nutritional status) and access to healthcare with the outcome. In addition to factors on the pathway to preterm birth, in view of the strong association between preterm birth and early child mortality, neonatal mortality rate will also be considered as a potential predictor of preterm birth in the statistical analysis. Table 2 presents candidate set of domains and variables from where potential covariates will be considered for inclusion in the statistical analysis.

**Table 2:** Covariates for potential inclusion in the modelling analyses.

Conceptual Domain	Potential Covariate
**Socio-economic, demographic, and cultural factors**	Gross National Income
	Adult Female Literacy Rate
	Adolescent Fertility Rate
	Total Fertility Rate
	Urban Population
**Nutritional, behavioral, and environmental factors**	Adult Female Smoking Rate
	Air Pollution
	Adult Female Body Mass Index
	Maternal Anemia
	Adult Female Substance Use
	Intimate Partner Violence
**Maternal conditions (including infections)**	Adult Female HIV Prevalence
	Malaria Incidence (*P*. *falciparum *Parasite Rate)
	Gestational Hypertension
	Gestational Diabetes
	Maternal Depression
**Fetal conditions**	Twinning
	Birth Defects
	Growth restriction
**Healthcare-related factors**	Antenatal Care Attendance (Four or more times)
	Skilled Birth Attendance
	Facility Birth Rate
	Cesarean Section Rate
**Other associated factors**	Neonatal Mortality Rate
	Geographical Region (UN M49 Regional Classification)

The covariate time-series data will include a well-documented methodology on construction of the time-series data and handling of missing covariate data across countries and years. We will explore methods for handling missing data such as; interpolation (if the covariate measure is such that it can reasonably be expected to change linearly over short time intervals), replacing with the nearest available value (if the nature of the construct it measures is such that it stays constant over moderate intervals and then changes abruptly) or, multiple imputation (if the missing values can be reasonably considered to be missing at random). Covariates with high degree of missing data or if uncertainty exists will be excluded.

#### Step 2: Modelling to estimate preterm birth rates

The modelling will follow a similar strategy to that applied for the previous preterm estimates i.e., using a Bayesian multilevel-mixed regression model [21]. The model will process population standardized preterm birth data of all country-years with ‘available data’, including the UN regional [37] intercepts and random country-specific intercepts and slopes, generating preterm birth estimates for these country-year points. The model will include year (time), data source characteristics and covariates. Due to large differences in sizes between administrative data and research studies, no weight for sample size will be used in the analysis. Linear relationships between preterm birth rates over time and for covariates will be assessed graphically and if non-linear, appropriate transformations e.g., spline functions, or fractional polynomials will be considered. During this analytical process, regional models will only be used on available data and in next step; preterm birth rates will be imputed for missing country-year records.

#### Step 3: Generating estimates of preterm birth rate

Estimates of preterm birth at global, regional, and national levels for 2010–2019 will be predicted from the models in step 2 using a Bayesian multilevel-mixed regression model (region, country and preterm birth rates). Fitted estimates generated from the model that includes data on year (time), source characteristics, and covariate data will be used to predict estimates for country-years with no available data. The Markov chain Monte Carlo method will be used to estimate the preterm birth rates based on priors (e.g., defining priors based on previous preterm estimates and other sources will be considered), data inputs, and covariate information to generate predictions for all country-years from the estimated model parameters. Logit or log transformations will be applied, as appropriate, to ensure that preterm birth estimates obtained from the fitted model are within a plausible range i.e., 0% and 100%.

#### Step 4: Presentation of results and sensitivity analysis

Country level point estimates with the 10^th^ and 90^th^ percentiles for uncertainty intervals around the estimate will be presented. We plan to conduct sensitivity analyses on the preterm birth outcome according to the risks of bias assessed (**Table 1**). We will publish national-level preterm birth rates for those countries with at least one eligible data point included in the estimation period, although all country-level estimates will contribute to the overall global and regional preterm birth rate estimates.

### Methodological limitations

There are several methodological challenges in the development of global preterm birth estimates that are anticipated in this work and should be noted as part of the study protocol.

First, national administrative data sources are often incomplete or unavailable for many countries, particularly low- and middle-income countries (LMICs). National administrative data sources, may also exclude marginalized or vulnerable groups (e.g. humanitarian settings, indigenous populations) who may face greater risks of preterm birth. Given the paucity of data from national administrative sources, we will use research studies from sub-national areas or populations as a supplemental data source. Research studies may have their own potential biases since they are often conducted at tertiary settings and/or in special population subgroups (for example; women with pre-eclampsia only) and may not accurately reflect the composition of the national population.

Second, gestational age measurement to classify a preterm birth is limited in many countries, and even when assessed and recorded during pregnancy, may not be aggregated into routine data systems. This is a considerable limitation affecting data availability from national administrative data sources for this estimation work. Access to early pregnancy ultrasound is limited across LMICs, and less accurate measures are commonly used as alternatives, such as last menstrual period, symphysis-fundal height assessment, or ultrasound scan in later trimesters that may lead to biases in either direction. Differences in gestational age measurements used to define preterm birth will also affect comparisons over time or across countries. Many national reports may also not specify the method of gestational age measurement used to define preterm birth, making interpretation of national results and comparisons across countries or over time more difficult.

Third, different definitions of preterm birth may be used across settings complicating cross-country comparisons (e.g. differences in denominator, gestational age ranges to define preterm birth, viability thresholds).

Fourth, the preterm birth definition used in this study includes only newborns who are born alive and preterm, which will underestimate the burden of prematurity as a public health issue since stillbirths are not included. In addition, misclassification of live preterm births who die shortly after birth as stillbirths can occur, this is most common around the thresholds of viability in all contexts, where signs of life may be harder to identify, and resuscitation may not be attempted While this current round of preterm birth estimates does not include stillbirths in the definition, work is currently underway to develop methods to include stillbirths in future estimation rounds.

## Discussion

The work described in this protocol aims to generate estimates of preterm birth rates at global, regional, and national levels for the period 2010 to 2019, which builds closely on the methodology used in the previous estimation rounds. In each successive estimation round, improvements in administrative data systems allow for an expanded number of national data points to be included in the estimation work in order to improve overall estimates. Some national household surveys are also initiating data collection on women who received an ultrasound during pregnancy, which could further expand data availability for this estimation round and going forward [38].

The current round of preterm birth estimates for the period 2010 to 2019 are the first to cover the beginning of the SDG era, and will inform the Survive, Thrive, Transform agenda of the Global Strategy for Women’s, Children’s and Adolescents’ Health (2016–2030) [22]. Up-to-date global, regional and national preterm birth estimates are critical for targeting programs that aim to reduce preterm birth rates over time. More recent estimates will also aid the development and implementation of health policies, inform resource allocation within health systems, and may be used to assess the impact of newborn survival interventions.

### Ethics and dissemination

#### Research ethics approval

Not applicable. This work is based on secondary analyses of public data from administrative sources and studies identified through a systematic search and review process.

#### Access to data

In compliance with GATHER guidelines [39], the final preterm birth estimates with uncertainty intervals will be published online through the WHO Global Health Observatory and UNICEF websites alongside the complete database of input data used to develop modelled estimates and relevant code. The following information will be made publicly available for all included data sources: reference information or contact name/institution, population represented, data collection method, year(s) of data collection, gestational age measurement method, and sample size as relevant.

#### Dissemination policy

This work will result in publication of global, regional and national preterm birth estimates for the period 2010–2019 in an open-access peer-reviewed journal. We will also publish the final protocol, database and preterm birth estimates online through the WHO Global Health Observatory and UNICEF websites according to GATHER guidelines [39], as described in the previous section.

### Administrative information

#### Registration

PROSPERO registration number CRD42021237861.

#### Protocol version and update

Protocol date: 4th June 2021; Protocol version: v.20. This protocol is not an amendment of a previously completed or published protocol. Any important protocol amendments will be documented in a protocol addendum and in the final report or manuscript.

## Supporting information

S1 Appendix Comparison of different methods for gestational age measurement [40]. (DOCX)

S2 Appendix Search strategy used to identify research studies. (DOCX)

S3 Appendix Data extraction form for low birthweight and preterm birth estimates. (XLS)
