# Supplementary material for: Study protocol for WHO and UNICEF estimates of global, regional, and national preterm birth rates for 2010 to 2019
Source: PLoS One. 2021 Oct 20;16(10):e0258751. doi: 10.1371/journal.pone.0258751 (PMC8528299; doi:10.1371/journal.pone.0258751)
Supplement: S1 Appendix — (DOCX) [file pone.0258751.s001.docx]

**S1 Appendix. Comparison of different methods for gestational age measurement**

| **Timing** | **Method** | **Accuracy** |
| --- | --- | --- |
| At any time | Last Menstrual Period | +/- 2 – 3 weeks (accuracy strongly affected by individual woman factors and whether collected prospectively or retrospectively) |
| Before birth only | Ultrasound fetal measurements ** | +/- 5 – 21 days (depending on gestational age when performed. More accurate when measurements taken at earlier gestations) |
| Before birth only | Symphysis Fundal Height | +/- 2 - 3 weeks (depending on gestational age when performed. More accurate when measurements taken at earlier gestations) |
| After birth only | New-born Clinical gestational age assessment scores | +/- 2 – 4 weeks (depending on tool used)  Assessment not possible in stillborn or very sick newborns who die soon after birth |
| After birth only | New-born anthropometric proxies | Variable depending on cut offs used. |
| After birth only | New-born assessment of anterior capsule of the lens | Variable. Overall correlation with gestational age moderate. More accurate in low birthweight populations, even if growth restricted. Only useful for gestational age assessment up to 35 weeks |

^**^ It is possible to combine ultrasound fetal measurements with last menstrual period estimations using algorithms to generate a ‘Best Obstetric Estimate’ (40)
