# Supplementary material for: Study protocol for WHO and UNICEF estimates of global, regional, and national preterm birth rates for 2010 to 2019
Source: PLoS One. 2021 Oct 20;16(10):e0258751. doi: 10.1371/journal.pone.0258751 (PMC8528299; doi:10.1371/journal.pone.0258751)
Supplement: S2 Appendix — (DOCX) [file pone.0258751.s002.docx]

**EMBASE**

1. EMBASE; (Afghanistan OR Albania OR Algeria OR Angola OR Antigua OR Barbuda OR Argentina OR Armenia OR Armenian OR Aruba OR Azerbaijan OR Bahamas OR Barbados OR Bangladesh OR Benin OR Byelarus OR Byelorussian OR Belarus OR Belorussian OR Belorussia OR Belize OR Bhutan OR Bolivia OR Bosnia OR Herzegovina OR Hercegovina OR Botswana OR Brasil OR Brazil OR Bulgaria OR "Burkina Faso" OR "Burkina Fasso" OR "Upper Volta" OR Brunei OR Burundi OR Urundi OR Cambodia OR "Khmer Republic" OR Kampuchea OR Cameroon OR Cameroons OR Cameron OR Camerons OR "Cape Verde" OR "Cabo Verde" OR "Central African Republic" OR Chad OR Chile OR China OR Colombia OR Comoros OR "Comoro Islands" OR Comores OR Mayotte OR "Cook Islands" OR Congo OR Zaire OR "Costa Rica" OR "Cote d'Ivoire" OR "Ivory Coast" OR Cuba OR Cyprus OR Czechoslovakia OR "Czech Republic" OR Slovakia OR "Slovak Republic" OR Djibouti OR "French Somaliland" OR Dominica OR "Dominican Republic" OR "East Timor" OR "East Timur" OR "Timor Leste" OR Ecuador OR Egypt OR "United Arab Republic" OR "United Arab Emirates" OR "El Salvador" OR Eritrea OR Ethiopia OR Fiji OR France OR Gabon OR "Gabonese Republic" OR Gambia OR Gaza OR "Georgia Republic" OR "Georgian Republic" OR Ghana OR "Gold Coast" OR Grenada OR Guatemala OR Guinea OR Guam OR Guiana OR Guyana OR Haiti OR Honduras OR India OR Maldives OR Indonesia OR Italy OR Iran OR Iraq OR "Isle of Man" OR Jamaica OR Jordan OR Kazakhstan OR Kazakh OR Kenya OR Kiribati OR Korea OR Kosovo OR Kyrgyzstan OR Kirghizia OR "Kyrgyz Republic" OR Kirghiz OR Kirgizstan OR "Lao PDR" OR Laos OR Latvia OR Lebanon OR Lesotho OR Basutoland OR Liberia OR Libya OR Lithunania OR Luxemburg OR Luxembourg OR Macedonia OR Madagascar OR Malagasy OR Malaysia OR Malaya OR Malay OR Sabah OR Sarawak OR Malawi OR Nyasaland OR Mali OR Malta OR "Marshall Islands" OR Mauritania OR Mauritius OR "Agalega Islands" OR Mexico OR Micronesia OR "Middle East" OR Moldova OR Moldovia OR Moldovian OR Monaco OR Mongolia OR Montenegro OR Morocco OR Ifni OR Mozambique OR Myanmar OR Myanma OR Burma OR Namibia OR Nauru OR Nepal OR "Netherlands Antilles" OR "New Caledonia" OR "New Zealand" OR Nicaragua OR Niue OR Niger OR Nigeria OR "Northern Mariana Islands" OR Muscat OR Pakistan OR Palau OR Palestine OR Panama OR Paraguay OR Peru OR Philipines OR Phillipines OR Phillippines OR "Puerto Rico" OR Qatar OR Romania OR Rumania OR Roumania OR Russia OR Russian OR Rwanda OR Ruanda OR "Saint Kitts" OR "St Kitts" OR Nevis OR "Saint Lucia" OR "St Lucia" OR "Saint Vincent" OR "St Vincent" OR Grenadines OR Samoa OR "Samoan Islands" OR "Navigator Islands" OR "Navigator Island" OR "Sao Tome" OR "San Marino" OR "Saudi Arabia" OR Senegal OR Serbia OR Montenegro OR Seychelles OR "Sierra Leone" OR Slovenia OR "Sri Lanka" OR Ceylon OR "Solomon Islands" OR Somalia OR "South Africa" OR Sudan OR Suriname OR Surinam OR Swaziland OR Syria OR Tajikistan OR Tadzhikistan OR Tadjikistan OR Tadzhik OR Tanzania OR Thailand OR Togo OR "Togolese Republic" OR Tonga OR Trinidad OR Tobago OR Tunisia OR Turkey OR Turkmenistan OR Turkmen OR Uganda OR Ukraine OR Uruguay OR USSR OR Switzerland OR "Syrian Arab Republic" OR "Soviet Union" OR "Union of Soviet Socialist Republics" OR Uzbekistan OR Uzbek OR Vanatu OR "New Hebrides" OR Venezuela OR Vietnam OR "Viet Nam" OR "West Bank" OR Yemen OR Yugoslavia OR Zambia OR Zimbabwe OR Rhodesia).hw,ti,ab,cp;
2. EMBASE; ((developing OR "less developed" OR "lesser developed" OR "under developed" OR underdeveloped OR "middle income" OR "low income" OR "lower income" OR underserved OR "under served" OR deprived OR poor*) ADJ (economy OR economies)).ti,ab;
3. EMBASE; ((developing OR "less developed" OR "lesser developed" OR "under developed" OR underdeveloped OR "middle income" OR "low income" OR "lower income" OR underserved OR "under served" OR deprived OR poor*) ADJ (countr* OR nation? OR population? OR world)).ti,ab;
4. EMBASE; (low* ADJ (gdp OR gnp OR "gross domestic" OR "gross national")).ti,ab;
5. EMBASE; (low ADJ middle adj3 countr*).ti,ab; 314 results.
6. EMBASE; (lmic OR lmics OR "third world" OR "lami country" OR "lami countries").ti,ab;
7. EMBASE; ("transitional country" OR "transitional countries").ti,ab;
8. EMBASE; (Africa OR Asia OR Caribbean OR "West Indies" OR "South America" OR "Latin America" OR

“Central America").hw,ti,ab,cp;

1. EMBASE; exp DEVELOPING COUNTRY/;
2. EMBASE; exp PREMATURE LABOR/;
3. EMBASE; (preterm OR pre-term OR "pre term" OR premature OR pre-mature OR "pre mature" OR prematuritas OR prematurity OR pre-maturity OR "pre maturity").ti,ab;
4. EMBASE; (labour OR labor OR birth OR child-birth OR childbirth OR "child birth" OR delivery OR deliveries OR parturition).ti,ab;
5. EMBASE; 11 AND 12;
6. EMBASE; 10 OR 13;
7. EMBASE; pprom.ti,ab;
8. EMBASE; exp PREMATURE FETUS MEMBRANE RUPTURE/;
9. EMBASE; ((prelabor OR pre-labor OR "pre labor" OR preterm OR pre-term OR "pre term" OR premature OR pre-mature OR "pre mature") adj3 (ruptur$ OR membrane$ OR ROM)).ti,ab;
10. EMBASE; 15 OR 16 OR 17;
11. EMBASE; 14 OR 18;
12. EMBASE; 1 OR 2 OR 3 OR 4 OR 5 OR 6 OR 7 OR 8 OR 9;
13. EMBASE; 19 AND 20;

22. EMBASE; 21 [Limit to: (Records From Embase) and Publication Year XXXX-YYYY]

**MEDLINE**

1. exp Premature Birth/
2. exp Obstetric Labor, Premature/
3. (preterm or pre-term or "pre term" or premature or pre-mature or "pre mature" or prematuritas or prematurity or pre-maturity or "pre maturity").ti,ab.
4. (labour or labor or birth or child-birth or childbirth or "child birth" or delivery or deliveries or parturition).ti,ab.
5. 3 and 4
6. 1 or 2 or 5
7. exp Fetal Membranes, Premature Rupture/
8. ((prelabor or pre-labor or "pre labor" or preterm or pre-term or "pre term" or premature or pre- mature or "pre mature") adj3 (ruptur$ or membrane$ or ROM)).ti,ab.
9. pprom.ti,ab.
10. 7 or 8 or 9
11. 6 or 10
12. developing countries.sh,kf.
13. (africa or asia or caribbean or west indies or south america or central america).hw,kf,ti,ab,cp
14. ("Lao PDR" or Laos or Latvia or Lebanon or Lesotho or Basutoland or Liberia or Libya or Lithunania or Luxemburg or Luxembourg or Monaco or Macedonia or Madagascar or Malagasy or Malaysia or Malaya or Malay or Sabah or Sarawak or Malawi or Nyasaland or Mali or Malta or "Marshall Islands" or Mauritania or Mauritius or "Agalega Islands" or Mexico or Micronesia or "Middle East" or Moldova or Moldovia or Moldovian or Mongolia or Montenegro or Morocco or Ifni or Mozambique or Myanmar or Myanma or Burma or Nauru or Niue or "New Zealand" or Namibia or Nepal or "Netherlands Antilles" or "New Caledonia" or Nicaragua or Niger or Nigeria or "Northern Mariana Islands" or Muscat or Pakistan or Palau or Palestine or Panama or Paraguay or Peru or Philipines or Phillipines or Phillippines or "Puerto Rico" or Qatar or Romania or Rumania or Roumania or Russia or Russian or Rwanda or Ruanda or "Saint Kitts" or "St Kitts" or Nevis or "Saint Lucia" or "St Lucia" or "Saint Vincent" or "St Vincent" or Grenadines or Samoa or "Samoan Islands" or "Navigator Islands" or "Navigator Island" or "Sao Tome" or "Saudi Arabia" or Senegal or Serbia or Montenegro or Seychelles or "Sierra Leone").hw,kf,ti,ab,cp.
15. (Slovenia or "Sri Lanka" or Ceylon or Switzerland or "Syrian Arab Republic" or "San Marino" or "Solomon Islands" or Somalia or "South Africa" or Sudan or Suriname or Surinam or Swaziland or Syria or Tajikistan or Tadzhikistan or Tadjikistan or Tadzhik or Tanzania or Thailand or Togo or "Togolese Republic" or Tonga or Trinidad or Tobago or Tunisia or Turkey or Turkmenistan or Turkmen or Uganda or Ukraine or Uruguay or USSR or "Soviet Union" or "Union of Soviet Socialist Republics" or Uzbekistan or Uzbek or Vanatu or "New Hebrides" or Venezuela or Vietnam or "Viet Nam" or "West Bank" or Yemen or Yugoslavia or Zambia or Zimbabwe or Rhodesia).hw,kf,ti,ab,cp.
16. ((developing or less* developed or under developed under developed or middle income or low* income or underserved or under served or deprived or poor*) adj (countr* or nation? or population? or world)).ti,ab.
17. ((developing or less* developed or under developed or underdeveloped or middle income or low* income) adj (economy or economies)).ti,ab.
18. (low* adj (gdp or gnp or gross domestic or gross national)).ti,ab.
19. (low adj middle adj3 countr*).ti,ab.
20. (lmic or lmics or third world or lami countri*).ti,ab.
21. Transitional countr*.ti,ab
22. (Afghanistan or Albania or Algeria or Angola or Antigua or Barbuda or Argentina or Armenia or Armenian or Aruba or Azerbaijan or Bangladesh or Bahamas or Brunei or Barbados or Benin or Byelarus or Byelorussian or Belarus or Belorussian or Belorussia or Belize or Bhutan or Bolivia or Bosnia or Herzegovina or Hercegovina or Botswana or Brasil or Brazil or Bulgaria or "Burkina Faso" or "Burkina Fasso" or "Upper Volta" or Burundi or Urundi or Cambodia or "Khmer Republic" or Kampuchea or Cameroon or Cameroons or Cameron or Camerons or "Cape Verde" or "Capo Verde" or "Central African Republic" or Chad or Chile or China or Colombia or Comoros or "Comoro Islands" or Comores or Mayotte or Congo or Zaire or "Costa Rica" or "Cote d'Ivoire" or "Ivory Coast" or Cuba or Cyprus or Djibouti or Czechoslovakia or "Czech Republic" or Slovakia or "Slovak Republic" or "French Somaliland" or Dominica or "Dominican Republic" or "East Timor" or "East Timur" or "Timor Leste" or Ecuador or Egypt or "United Arab Republic" or "United Arab Emirates" or "El Salvador" or Eritrea or Ethiopia or France or Fiji or Gabon or "Gabonese Republic" or Gambia or Gaza or "Georgia Republic" or "Georgian Republic" or Ghana or "Gold Coast" or Grenada or Guatemala or Guinea or Guam or Guiana or Guyana or Haiti or Honduras or India or Italy or Maldives or Indonesia or Iran or Iraq or "Isle of Man" or Italy or Jamaica or Jordan or Kazakhstan or Kazakh or Kenya or Kiribati or Korea or Kosovo or Kyrgyzstan or Kirghizia or "Kyrgyz Republic" or Kirghiz or Kirgizstan).hw,kf,ti,ab,cp.
23. 12 or 13 or 14 or 15 or 16 or 17 or 18 or 19 or 20 or 21 or 22
24. 11 and 23
25. Global Health/sn [Statistics & Numerical Data]
26. 11 and 25
27. 24 or 26
28. 28. limit 27 to yr="XXXX-YYYY"

**POPLINE**

Afghanistan OR Albania OR Algeria OR Angola OR Antigua OR Barbuda OR Argentina OR Armenia OR Armenian OR Aruba OR Azerbaijan OR Bahamas OR Barbados OR Bangladesh OR Benin OR Byelarus OR Byelorussian OR Belarus OR Belorussian OR Belorussia OR Belize OR Bhutan OR Bolivia OR Bosnia OR Herzegovina OR Hercegovina OR Botswana OR Brasil OR Brazil OR Bulgaria OR "Burkina Faso" OR "Burkina Fasso" OR "Upper Volta" OR Brunei OR Burundi OR Urundi OR Cambodia OR "Khmer Republic" OR Kampuchea OR Cameroon OR Cameroons OR Cameron OR Camerons OR "Cape Verde" OR "Cabo Verde" OR "Central African Republic" OR Chad OR Chile OR China OR Colombia OR Comoros OR "Comoro Islands" OR Comores OR Mayotte OR "Cook Islands" OR Congo OR Zaire OR "Costa Rica" OR "Cote d'Ivoire" OR "Ivory Coast" OR Cuba OR Cyprus OR Czechoslovakia OR "Czech Republic" OR Slovakia OR "Slovak Republic" OR Djibouti OR "French Somaliland" OR Dominica OR "Dominican Republic" OR "East Timor" OR "East Timur" OR "Timor Leste" OR Ecuador OR Egypt OR "United Arab Republic" OR "United Arab Emirates" OR "El Salvador" OR Eritrea OR Ethiopia OR Fiji OR France OR Gabon OR "Gabonese Republic" OR Gambia OR Gaza OR "Georgia Republic" OR "Georgian Republic" OR Ghana OR "Gold Coast" OR Grenada OR Guatemala OR Guinea OR Guam OR Guiana OR Guyana OR Haiti OR Honduras OR India OR Maldives OR Indonesia OR Italy OR Iran OR Iraq OR "Isle of Man" OR Jamaica OR Jordan OR Kazakhstan OR Kazakh OR Kenya OR Kiribati OR Korea OR Kosovo OR Kyrgyzstan OR Kirghizia OR "Kyrgyz Republic" OR Kirghiz OR Kirgizstan OR "Lao PDR" OR Laos OR Latvia OR Lebanon OR Lesotho OR Basutoland OR Liberia OR Libya OR Lithunania OR Luxemburg OR Luxembourg OR Macedonia OR Madagascar OR Malagasy OR Malaysia OR Malaya OR Malay OR Sabah OR Sarawak OR Malawi OR Nyasaland OR Mali OR Malta OR "Marshall Islands" OR Mauritania OR Mauritius OR "Agalega Islands" OR Mexico OR Micronesia OR "Middle East" OR Moldova OR Moldovia OR Moldovian OR Monaco OR Mongolia OR Montenegro OR Morocco OR Ifni OR Mozambique OR Myanmar OR Myanma OR Burma OR Namibia OR Nauru OR Nepal OR "Netherlands Antilles" OR "New Caledonia" OR "New Zealand" OR Nicaragua OR Niue OR Niger OR Nigeria OR "Northern Mariana Islands" OR Muscat OR Pakistan OR Palau OR Palestine OR Panama OR Paraguay OR Peru OR Philipines OR Phillipines OR Phillippines OR "Puerto Rico" OR Qatar OR Romania OR Rumania OR Roumania OR Russia OR Russian OR Rwanda OR Ruanda OR "Saint Kitts" OR "St Kitts" OR Nevis OR "Saint Lucia" OR "St Lucia" OR "Saint Vincent" OR "St Vincent" OR Grenadines OR Samoa OR "Samoan Islands" OR "Navigator Islands" OR "Navigator Island" OR "Sao Tome" OR "San Marino" OR "Saudi Arabia" OR Senegal OR Serbia OR Montenegro OR Seychelles OR "Sierra Leone" OR Slovenia OR "Sri Lanka" OR Ceylon OR "Solomon Islands" OR Somalia OR "South Africa" OR Sudan OR Suriname OR Surinam OR Swaziland OR Syria OR Tajikistan OR Tadzhikistan OR Tadjikistan OR Tadzhik OR Tanzania OR Thailand OR Togo OR "Togolese Republic" OR Tonga OR Trinidad OR Tobago OR Tunisia OR Turkey OR Turkmenistan OR Turkmen OR Uganda OR Ukraine OR Uruguay OR USSR OR Switzerland OR "Syrian Arab Republic" OR "Soviet Union" OR "Union of Soviet Socialist Republics" OR Uzbekistan OR Uzbek OR Vanatu OR "New Hebrides" OR Venezuela OR Vietnam OR "Viet Nam" OR "West Bank" OR Yemen OR Yugoslavia OR Zambia OR Zimbabwe OR Rhodesia OR lmic OR lami OR "developing country OR "developing countries" OR "lower income country" OR "lower income countries" OR "middle income countries" OR middle income country" OR Africa OR Asia OR Caribbean OR "West Indies" OR "South America" OR "Latin America" OR "Central America”

AND

preterm OR premature or "pre term" OR pre-term OR "pre mature" OR pre-mature OR prematurity OR prelabour OR pre-labor OR prelabor OR prelabour OR "pre labour" OR "pre labor"

AND

labour OR labor OR birth OR childbirth OR child-birth OR "child birth" OR delivery OR deliveries OR parturition OR "ruptured membranes" OR "rupture of membranes" OR "rupture of the membranes" OR "membrane rupture"

**WHO GLOBAL HEALTH LIBRARY (including regional and global indexes)**

(tw:((rupture OR ruptured) AND (membranas OR membranes OR membrane) AND (pré-termo OR prematuro OR prematuros OR prematuridade OR prematuridad OR pre-termino OR prelabor OR pre-labor OR "pre labor" OR pre-labour OR prelabour OR "pre labour" OR preterm OR pre-term OR "pre term" OR premature OR pre- mature OR "pre mature"))) OR (tw:((labour OR labor OR parto OR birth OR child-birth OR childbirth OR "child birth" OR delivery OR deliveries OR parturition OR nascimento OR nascimentos OR nacimiento OR nacimientos) AND (pré-termo OR prematuro OR prematuros OR prematuridade OR prematuridad OR pre- termino OR prelabor OR pre-labor OR "pre labor" OR pre-labour OR prelabour OR "pre labour" OR preterm OR pre-term OR "pre term" OR premature OR pre-mature OR "pre mature")))

(tw:((rupture OR ruptured) AND (membranas OR membranes OR membrane) AND (pré-termo OR prematuro OR prematuros OR prematuridade OR prematuridad OR pre-termino OR prelabor OR pre-labor OR "pre labor" OR pre-labour OR prelabour OR "pre labour" OR preterm OR pre-term OR "pre term" OR premature OR pre- mature OR "pre mature"))) OR (tw:((labour OR labor OR parto OR birth OR child-birth OR childbirth OR "child birth" OR delivery OR deliveries OR parturition OR nascimento OR nascimentos OR nacimiento OR nacimientos) AND (pré-termo OR prematuro OR prematuros OR prematuridade OR prematuridad OR pre- termino OR prelabor OR pre-labor OR "pre labor" OR pre-labour OR prelabour OR "pre labour" OR preterm OR pre-term OR "pre term" OR premature OR pre-mature OR "pre mature")))

**CINAHL**

1. CINAHL; (preterm OR pre-term OR "pre term" OR premature OR pre-mature OR "pre mature" OR prematuritas OR prematurity OR pre-maturity OR "pre maturity").ti,ab; 21432 results.
2. CINAHL; (labour OR labor OR birth OR child-birth OR childbirth OR "child birth" OR delivery OR deliveries OR parturition).ti,ab; 90297 results.
3. CINAHL; 1 AND 2; 9653 results.
4. CINAHL; (prelabor OR pre-labor OR "pre labor" OR preterm OR pre-term OR "pre term" OR premature OR pre-mature OR "pre mature").ti,ab; 20403 results.
5. CINAHL; (ruptur$ OR membrane$ OR ROM).ti,ab; 12032 results.
6. CINAHL; 4 AND 5; 831 results.
7. CINAHL; pprom.ti,ab; 148 results.
8. CINAHL; exp LABOR, PREMATURE/; 2092 results.
9. CINAHL; exp CHILDBIRTH, PREMATURE/; 4436 results.
10. CINAHL; exp FETAL MEMBRANES, PREMATURE RUPTURE/; 1331 results.
11. CINAHL; 3 OR 6 OR 7 OR 8 OR 9 OR 10; 13016 results.
12. CINAHL; (Afghanistan OR Albania OR Algeria OR Angola OR Antigua OR Barbuda OR Argentina OR Armenia OR Armenian OR Aruba OR Azerbaijan OR Bahamas OR Barbados OR Bangladesh OR Benin OR Byelarus OR Byelorussian OR Belarus OR Belorussian OR Belorussia OR Belize OR Bhutan OR Bolivia OR Bosnia OR Herzegovina OR Hercegovina OR Botswana OR Brasil OR Brazil OR Bulgaria OR "Burkina Faso" OR "Burkina Fasso" OR "Upper Volta" OR Brunei OR Burundi OR Urundi OR Cambodia OR "Khmer Republic" OR Kampuchea OR Cameroon OR Cameroons OR Cameron OR Camerons OR "Cape Verde" OR "Cabo Verde" OR "Central African Republic" OR Chad OR Chile OR China OR Colombia OR Comoros OR "Comoro Islands" OR Comores OR Mayotte OR "Cook Islands" OR Congo OR Zaire OR "Costa Rica" OR "Cote d'Ivoire" OR "Ivory Coast" OR Cuba OR Cyprus OR Czechoslovakia OR "Czech Republic" OR Slovakia OR "Slovak Republic" OR Djibouti OR "French Somaliland" OR Dominica OR "Dominican Republic" OR "East Timor" OR "East Timur" OR "Timor Leste" OR Ecuador OR Egypt OR "United Arab Republic" OR "United Arab Emirates" OR "El Salvador" OR Eritrea OR Ethiopia OR Fiji OR France OR Gabon OR "Gabonese Republic" OR Gambia OR Gaza OR "Georgia Republic" OR "Georgian Republic" OR Ghana OR "Gold Coast" OR Grenada OR Guatemala OR Guinea OR Guam OR Guiana OR Guyana OR Haiti OR Honduras OR India OR Maldives OR Indonesia OR Italy OR Iran OR Iraq OR "Isle of Man" OR Jamaica OR Jordan OR Kazakhstan OR Kazakh OR Kenya OR Kiribati OR Korea OR Kosovo OR Kyrgyzstan OR Kirghizia OR "Kyrgyz Republic" OR Kirghiz OR Kirgizstan OR "Lao PDR" OR Laos OR Latvia OR Lebanon OR Lesotho OR Basutoland OR Liberia OR Libya OR Lithunania OR Luxemburg OR Luxembourg OR Macedonia OR Madagascar OR Malagasy OR Malaysia OR Malaya OR Malay OR Sabah OR Sarawak OR Malawi OR Nyasaland OR Mali OR Malta OR "Marshall Islands" OR Mauritania OR Mauritius OR "Agalega Islands" OR Mexico OR Micronesia OR "Middle East" OR Moldova OR Moldovia OR Moldovian OR Monaco OR Mongolia OR Montenegro OR Morocco OR Ifni OR Mozambique OR Myanmar OR Myanma OR Burma OR Namibia OR Nauru OR Nepal OR "Netherlands Antilles" OR "New Caledonia" OR "New Zealand" OR Nicaragua OR Niue OR Niger OR Nigeria OR "Northern Mariana Islands" OR Muscat OR Pakistan OR Palau OR Palestine OR Panama OR Paraguay OR Peru OR Philipines OR Phillipines OR Phillippines OR "Puerto Rico" OR Qatar OR Romania OR Rumania OR Roumania OR Russia OR Russian OR Rwanda OR Ruanda OR "Saint Kitts" OR "St Kitts" OR Nevis OR "Saint Lucia" OR "St Lucia" OR "Saint Vincent" OR "St Vincent" OR Grenadines OR Samoa OR "Samoan Islands" OR "Navigator Islands" OR "Navigator Island" OR "Sao Tome" OR "San Marino" OR "Saudi Arabia" OR Senegal OR Serbia OR Montenegro OR Seychelles OR "Sierra Leone" OR Slovenia OR "Sri Lanka" OR Ceylon OR "Solomon Islands" OR Somalia OR "South Africa" OR Sudan OR Suriname OR Surinam OR Swaziland OR Syria OR Tajikistan OR Tadzhikistan OR Tadjikistan OR Tadzhik OR Tanzania OR Thailand OR Togo OR "Togolese Republic" OR Tonga OR Trinidad OR Tobago OR Tunisia OR Turkey OR Turkmenistan OR Turkmen OR Uganda OR Ukraine OR Uruguay OR USSR OR Switzerland OR "Syrian Arab Republic" OR "Soviet Union" OR "Union of Soviet Socialist Republics" OR Uzbekistan OR Uzbek OR Vanatu OR "New Hebrides" OR Venezuela OR Vietnam OR "Viet Nam" OR "West Bank" OR Yemen OR Yugoslavia OR Zambia OR Zimbabwe OR Rhodesia).hw,ti,ab,cp
13. CINAHL; ((developing OR "less developed" OR "lesser developed" OR "under developed" OR underdeveloped OR "middle income" OR "low income" OR "lower income" OR underserved OR "under served" OR deprived OR poor*) ADJ (economy OR economies)).ti,ab; 62 results.
14. CINAHL; ((developing OR "less developed" OR "lesser developed" OR "under developed" OR underdeveloped OR "middle income" OR "low income" OR "lower income" OR underserved OR "under served" OR deprived OR poor*) ADJ (countr* OR nation? OR population? OR world)).ti,ab; 9427 results.
15. CINAHL; (low* ADJ (gdp OR gnp OR "gross domestic" OR "gross national")).ti,ab; 16 results.
16. CINAHL; (low ADJ middle adj3 countr*).ti,ab; 59 results.
17. CINAHL; (lmic OR lmics OR "third world" OR "lami country" OR "lami countries").ti,ab; 494 results.
18. CINAHL; ("transitional country" OR "transitional countries").ti,ab; 32 results.
19. CINAHL; (Africa OR Asia OR Caribbean OR "West Indies" OR "South America" OR "Latin America" OR "Central America").hw,ti,ab,cp; 15160 results.
20. CINAHL; exp DEVELOPING COUNTRIES/; 8889 results.
21. CINAHL; 12 OR 13 OR 14 OR 15 OR 16 OR 17 OR 18 OR 19 OR 20; 108294 results.
22. CINAHL; 67 AND 21; 613 results.
23. CINAHL; 22 [Limit to: Publication Year 1990-2016]; 610 results.

**PSYCHINFO**

1. PsycInfo; (preterm OR pre-term OR "pre term" OR premature OR pre-mature OR "pre mature" OR prematuritas OR prematurity OR pre-maturity OR "pre maturity").ti,ab; 15687 results.
2. PsycInfo; (labour OR labor OR birth OR child-birth OR childbirth OR "child birth" OR delivery OR deliveries OR parturition).ti,ab; 110875 results.
3. PsycInfo; 1 AND 2; 4527 results.
4. PsycInfo; (prelabor OR pre-labor OR "pre labor" OR preterm OR pre-term OR "pre term" OR premature OR pre-mature OR "pre mature").ti,ab; 15055 results.
5. PsycInfo; (ruptur$ OR membrane$ OR ROM).ti,ab; 15379 results.
6. PsycInfo; 4 AND 5; 85 results.
7. PsycInfo; pprom.ti,ab; 4 results.
8. PsycInfo; exp PREMATURE BIRTH/; 4609 results.
9. PsycInfo; ((developing OR "less developed" OR "lesser developed" OR "under developed" OR underdeveloped OR "middle income" OR "low income" OR "lower income" OR underserved OR "under served" OR deprived OR poor*) ADJ (economy OR economies)).ti,ab; 234 results.
10. PsycInfo; ((developing OR "less developed" OR "lesser developed" OR "under developed" OR underdeveloped OR "middle income" OR "low income" OR "lower income" OR underserved OR "under served" OR deprived OR poor*) ADJ (countr* OR nation? OR population? OR world)).ti,ab; 7173 results.
11. PsycInfo; (low* ADJ (gdp OR gnp OR "gross domestic" OR "gross national")).ti,ab; 20 results.
12. PsycInfo; (low ADJ middle adj3 countr*).ti,ab; 174 results.
13. PsycInfo; (lmic OR lmics OR "third world" OR "lami country" OR "lami countries").ti,ab; 1185 results.
14. PsycInfo; ("transitional country" OR "transitional countries").ti,ab; 51 results.
15. PsycInfo; (Africa OR Asia OR Caribbean OR "West Indies" OR "South America" OR "Latin America" OR "Central America").ti,ab; 26321 results.
16. PsycInfo; exp DEVELOPING COUNTRIES/; 4542 results.
17. PsycInfo; (Afghanistan OR Albania OR Algeria OR Angola OR Antigua OR Barbuda OR Argentina OR Armenia OR Armenian OR Aruba OR Azerbaijan OR Bahamas OR Barbados OR Bangladesh OR Benin OR Byelarus OR Byelorussian OR Belarus OR Belorussian OR Belorussia OR Belize OR Bhutan OR Bolivia OR Bosnia OR Herzegovina OR Hercegovina OR Botswana OR Brasil OR Brazil OR Bulgaria OR "Burkina Faso" OR "Burkina Fasso" OR "Upper Volta" OR Brunei OR Burundi OR Urundi OR Cambodia OR "Khmer Republic" OR Kampuchea OR Cameroon OR Cameroons OR Cameron OR Camerons OR "Cape Verde" OR "Cabo Verde" OR "Central African Republic" OR Chad OR Chile OR China OR Colombia OR Comoros OR "Comoro Islands" OR Comores OR Mayotte OR "Cook Islands" OR Congo OR Zaire OR "Costa Rica" OR "Cote d'Ivoire" OR "Ivory Coast" OR Cuba OR Cyprus OR Czechoslovakia OR "Czech Republic" OR Slovakia OR "Slovak Republic" OR Djibouti OR "French Somaliland" OR Dominica OR "Dominican Republic" OR "East Timor" OR "East Timur" OR "Timor Leste" OR Ecuador OR Egypt OR "United Arab Republic" OR "United Arab Emirates" OR "El Salvador" OR Eritrea OR Ethiopia OR Fiji OR France OR Gabon OR "Gabonese Republic" OR Gambia OR Gaza OR "Georgia Republic" OR "Georgian Republic" OR Ghana OR "Gold Coast" OR Grenada OR Guatemala OR Guinea OR Guam OR Guiana OR Guyana OR Haiti OR Honduras OR India OR Maldives OR Indonesia OR Italy OR Iran OR Iraq OR "Isle of Man" OR Jamaica OR Jordan OR Kazakhstan OR Kazakh OR Kenya OR Kiribati OR Korea OR Kosovo OR Kyrgyzstan OR Kirghizia OR "Kyrgyz Republic" OR Kirghiz OR Kirgizstan OR "Lao PDR" OR Laos OR Latvia OR Lebanon OR Lesotho OR Basutoland OR Liberia OR Libya OR Lithunania OR Luxemburg OR Luxembourg OR Macedonia OR Madagascar OR Malagasy OR Malaysia OR Malaya OR Malay OR Sabah OR Sarawak OR Malawi OR Nyasaland OR Mali OR Malta OR "Marshall Islands" OR Mauritania OR Mauritius OR "Agalega Islands" OR Mexico OR Micronesia OR "Middle East" OR Moldova OR Moldovia OR Moldovian OR Monaco OR Mongolia OR Montenegro OR Morocco OR Ifni OR Mozambique OR Myanmar OR Myanma OR Burma OR Namibia OR Nauru OR Nepal OR "Netherlands Antilles" OR "New Caledonia" OR "New Zealand" OR Nicaragua OR Niue OR Niger OR Nigeria OR "Northern Mariana Islands" OR Muscat OR Pakistan OR Palau OR Palestine OR Panama OR Paraguay OR Peru OR Philipines OR Phillipines OR Phillippines OR "Puerto Rico" OR Qatar OR Romania OR Rumania OR Roumania OR Russia OR Russian OR Rwanda OR Ruanda OR "Saint Kitts" OR "St Kitts" OR Nevis OR "Saint Lucia" OR "St Lucia" OR "Saint Vincent" OR "St Vincent" OR Grenadines OR Samoa OR "Samoan Islands" OR "Navigator Islands" OR "Navigator Island" OR "Sao Tome" OR "San Marino" OR "Saudi Arabia" OR Senegal OR Serbia OR Montenegro OR Seychelles OR "Sierra Leone" OR Slovenia OR "Sri Lanka" OR Ceylon OR "Solomon Islands" OR Somalia OR "South Africa" OR Sudan OR Suriname OR Surinam OR Swaziland OR Syria OR Tajikistan OR Tadzhikistan OR Tadjikistan OR Tadzhik OR Tanzania OR Thailand OR Togo OR "Togolese Republic" OR Tonga OR Trinidad OR Tobago OR Tunisia OR Turkey OR Turkmenistan OR Turkmen OR Uganda OR Ukraine OR Uruguay OR USSR OR Switzerland OR "Syrian Arab Republic" OR "Soviet Union" OR "Union of Soviet Socialist Republics" OR Uzbekistan OR Uzbek OR Vanatu OR "New Hebrides" OR Venezuela OR Vietnam OR "Viet Nam" OR "West Bank" OR Yemen OR Yugoslavia OR Zambia OR Zimbabwe OR Rhodesia).ti,ab,su;
18. PsycInfo; 3 OR 6 OR 7 OR 8; 7011 results.
19. PsycInfo; 9 OR 10 OR 11 OR 12 OR 13 OR 14 OR 16 OR 17 OR 21; 317904 results.
20. PsycInfo; 22 AND 23; 730 results.
21. PsycInfo; 24 [Limit to: Publication Year 1990-2016]; 699 results.

**COCHRANE LIBRARY FOR SYSTEMATIC REVIEWS**

#1 (Africa or Asia or Caribbean or "West Indies" or "South America" or "Latin America" or "Central America"):ti,ab,kw

#2 (Afghanistan or Albania or Algeria or Angola or Antigua or Barbuda or Argentina or Armenia or Armenian or Aruba or Azerbaijan or Bahamas or Barbados or Bangladesh or Benin or Byelarus or Byelorussian or Belarus or Belorussian or Belorussia or Belize or Bhutan or Bolivia or Bosnia or Herzegovina or Hercegovina or Botswana or Brasil or Brazil or Brunei or Bulgaria or "Burkina Faso" or "Burkina Fasso" or "Upper Volta" or Burundi or Urundi or Cambodia or "Khmer Republic" or Kampuchea or Cameroon or Cameroons or Cameron or Camerons or "Cape Verde" or "Capo Verde" or "Central African Republic" or Chad or Chile or China or Colombia or Comoros or "Comoro Islands" or Comores or Mayotte or Congo or "Cook Islands" or Zaire or "Costa Rica" or "Cote d'Ivoire" or "Ivory Coast" or Cuba or Cyprus or Czechoslovakia or "Czech Republic OR Slovakia" or "Slovak Republic") .ti,ab,kw

#3 (Djibouti or "French Somaliland" or Dominica or "Dominican Republic" or "East Timor" or "East Timur" or "Timor Leste" or Ecuador or Egypt or "United Arab Republic" or "El Salvador" or Eritrea or Ethiopia or Fiji or France or Gabon or "Gabonese Republic" or Gambia or Gaza or Georgia or Georgian or Ghana or "Gold Coast" or Grenada or Guatemala or Guinea or Guam or Guiana or Guyana or Haiti or Honduras or India or Maldives or Indonesia or Iran or Iraq or "Isle of Man" or Italy or Jamaica or Jordan or Kazakhstan or Kazakh or Kenya or Kiribati or Korea or Kosovo or Kyrgyzstan or Kirghizia or "Kyrgyz Republic" or Kirghiz or Kirgizstan or "Lao PDR" or Laos or Latvia or Lebanon or Lesotho or Basutoland or Liberia or Libya or Lithuania or Luxembourg or Luxemburg):ti,ab,kw

#4 (Macedonia or Madagascar or "Malagasy Republic" or Malaysia or Malaya or Malay or Sabah or Sarawak or Malawi or Nyasaland or Mali or "Marshall Islands" or Mauritania or Mauritius or "Agalega Islands" or Mexico or Micronesia or "Middle East" or Moldova or Moldovia or Moldovian or Monaco or Mongolia or Montenegro or Morocco or Ifni or Mozambique or Myanmar or Myanma or Burma or Nauru or Namibia or Nepal or "Netherlands Antilles" or "New Caledonia" or "New Zealand" or Nieu or Nicaragua or Niger or Nigeria or "Northern Mariana Islands" or Muscat or Pakistan or Palau or Palestine or Panama or Paraguay or Peru or Philippines or Philipines or Phillipines or Phillippines or "Puerto Rico" or Qatar):ti,ab,kw

#5 (Romania or Rumania or Roumania or Russia or Russian or Rwanda or Ruanda or "Saint Kitts" or "St Kitts" or Nevis or "Saint Lucia" or "St Lucia" or "Saint Vincent" or "St Vincent" or Grenadines or Samoa or "Samoan Islands" or "Navigator Island" or "Navigator Islands" or "Sao Tome" or "San Marino" or "Saudi Arabia" or Senegal or Serbia or Montenegro or Seychelles or "Sierra Leone" or Slovenia or "Sri Lanka" or Ceylon or "Solomon Islands" or Somalia or Sudan or Suriname or Surinam or Swaziland or Switzerland or "Syrian Arab Republic" or Syria or Tajikistan or Tadzhikistan or Tadjikistan or Tadzhik or Tanzania or Thailand or Togo or "Togolese Republic" or Tonga or Trinidad or Tobago or Tunisia or Turkey or Turkmenistan or Turkmen or Uganda or Ukraine or Uruguay or USSR or "Soviet Union" or "Union of Soviet Socialist Republics" "United Arab Emirates" or Uzbekistan or Uzbek or Vanuatu or "New Hebrides" or Venezuela or Vietnam or "Viet Nam" or "West Bank" or Yemen or Yugoslavia or Zambia or Zimbabwe or Rhodesia):ti,ab,kw

#6 (developing or less* next developed or "under developed" or underdeveloped or "middle income" or low* next income or underserved or "under served" or deprived or poor*) next (countr* or nation* or population* or world):ti,ab,kw

#7 (developing or less* next developed or "under developed" or underdeveloped or "middle income" or low* next income) next (economy or economies):ti,ab,kw

#8 low* next (gdp or gnp or "gross domestic" or "gross national"):ti,ab,kw

#9 (low near/3 middle near/3 countr*):ti,ab,kw

#10 (lmic or lmics or "third world" or "lami country" or "lami countries"):ti,ab,kw

#11 ("transitional country" or "transitional countries"):ti,ab,kw

#12 (#1 or #2 or #3 or #4 or #5 or #6 or #7 or #8 or #9 or #10 or #11)

#13 (preterm near birth)

#14 (preterm near lab*r)

#15 (preterm near delivery)

#16 (pre-term near birth)

#17 (premature near birth)

#18 (premature near delivery)

#19 (premature near lab*r)

#20 MeSH descriptor: [Premature Birth] explode all trees

#21 MeSH descriptor: [Fetal Membranes, Premature Rupture] explode all trees

#22 prom or pprom

#23 membrane* and rupture* and preterm

#24 membrane* and rupture* and pre-term

#25 membrane* and rupture* and premature

#26 membrane* and rupture* and pre-labour

#27 membrane* and rupture* and prelabour

#28 membrane* and rupture* and pre-labor

#29 membrane* and rupture* and prelabor

#30 MeSH descriptor: [Obstetric Labor, Premature] explode all trees

#31 {or #13-#30}

#32 #12 and #31 Publication Year from 1990 to 2016

**SINOMED**

1. ((((("preterm birth") OR "spontaneous preterm birth") OR "preterm birth due to premature rupture of fetal membranes") OR "premature rupture of fetal membranes") OR "therapeutic preterm") OR " iatrogenic preterm labor ")
2. ("risk factors") OR ("incidence")
3. Publication Year：1990-2016
4. Publication Journals (Chinese Medical Journal) OR (Chinese Journal of Preventive Medicine) OR (Chinese Journal of Epidemiology) OR ( Chinese Journal of Obstetrics and Gynecology) OR ( Chinese Journal of Perinatal Medicine) OR (Chinese Journal of Pediatrics)
5. 1 and 2 and 3 (10509 results)
6. 4 and 5 (350 results)
